# Long-acting IL-7 induces distinct transcriptomic features in peripheral T cells of patients with solid tumors

**DOI:** 10.1172/jci.insight.203629

**Published:** 2026-04-21

**Authors:** Hocheol Jang, Jeong Yeon Kim, Sojeong Kim, Heewon Kim, Mi Sun Byun, Myung Ah Lee, Jong Hee Chang, Do-Hyun Nam, Tae Won Kim, Sin-Soo Jeun, Joo Hyuk Sohn, Su-Hyung Park, Eui-Cheol Shin

**Affiliations:** 1 Graduate School of Medical Science and Engineering, Korea Advanced Institute of Science and Technology (KAIST), Daejeon, Republic of Korea.; 2 Genexine, Inc., Seoul, Republic of Korea.; 3 Department of Internal Medicine, Seoul St. Mary’s Hospital, College of Medicine, The Catholic University, Seoul, Republic of Korea.; 4 Department of Neurosurgery, Yonsei University College of Medicine, Seoul, Republic of Korea.; 5 Department of Neurosurgery, Samsung Medical Center, Sungkyunkwan University School of Medicine, Seoul, Republic of Korea.; 6 Department of Oncology, Asan Medical Center, University of Ulsan College of Medicine, Seoul, Republic of Korea.; 7 Department of Neurosurgery, Seoul St. Mary’s Hospital, College of Medicine, The Catholic University of Korea, Seoul, Republic of Korea.; 8 Division of Medical Oncology, Department of Internal Medicine, Yonsei Cancer Center, Yonsei University College of Medicine, Seoul, Republic of Korea.

**Keywords:** Clinical Research, Immunology, Oncology, Cytokines, Immunotherapy, T cells

## Abstract

**BACKGROUND:**

IL-7 is a critical cytokine in T cell development, survival, and homeostasis. Previous preclinical and clinical studies reported that IL-7 treatment increased T cell counts, but its effect on peripheral blood T cells in cancer patients and molecular mechanisms have not been explored.

**METHODS:**

We investigated effects of long-acting recombinant human IL-7 conjugated to a hybrid IgD/IgG4 Fc domain (rhIL-7-hyFc) on peripheral T cells in patients with advanced solid tumors. Peripheral blood samples were collected before and after treatment, followed by analysis through single-cell transcriptomics and flow cytometry.

**RESULTS:**

We found that rhIL-7-hyFc induced marked expansion of proliferating T cells, and promoted transcriptional changes associated with immune activation, cell cycle progression, and antiapoptosis. Trajectory analysis revealed that posttreatment T cells had distinct transcriptional states enriched for cytokine- and TCR-mediated signaling pathways. Notably, a second dose administered after 3 weeks yielded diminished proliferation and minimal transcriptional changes, which were independent of antidrug antibody or CD127 downmodulation. Examination of elements of the IL-7 signaling pathway revealed intact proximal signaling (e.g., STAT5 phosphorylation) but downregulation of distal elements, including PIM-1 kinase and c-Myc.

**CONCLUSIONS:**

Our results demonstrate that rhIL-7-hyFc induces robust peripheral T cell expansion and activation in patients with solid tumors, supporting its potential use for lymphopenic patients treated with cancer immunotherapy.

**TRIAL REGISTRATION:**

ClinicalTrials.gov NCT03478995 and NCT03619239.

**FUNDING:**

National Research Foundation of Korea (NRF-2022R1A2C3007292 and RS-2024-00439160), Ministry of Food and Drug Safety (RS-2025-02213409), and the Korea Health Technology R&D Project through the Korea Health Industry Development Institute (RS-2025-25460003).

## Abstract 2

Administration of long-acting recombinant IL-7 induces distinct transcriptomic features in peripheral blood T cells of patients with solid tumors

## Introduction

T cells mediate antitumor immunity by recognizing and eliminating malignant cells (1–3). Cancer patients frequently exhibit T cell lymphopenia (a reduction in circulating T cell count), which correlates with impaired immune surveillance, reduced infiltration of effector cells into the tumor microenvironment, and weakened antitumor immunity (4). These effects contribute to poor clinical outcomes (5–10); thus, there is a need for strategies to restore T cell populations and enhance their function. One promising therapeutic approach involves interleukin-7 (IL-7), a cytokine primarily produced by stromal cells within the bone marrow and thymus that is required for T cell development, survival, and homeostasis (11–13). IL-7 binds to its cognate receptor complex comprising IL-7Rα and γc chains, and thereby initiates signaling that promotes T cell proliferation, enhances thymic output, and augments peripheral T cell survival (14, 15).

Preclinical and clinical studies have validated the therapeutic efficacy of IL-7 for reversing T cell lymphopenia (16, 17). In murine models of T cell lymphopenia induced by irradiation or chemotherapy, exogenous IL-7 administration stimulates thymic regeneration, enhances peripheral T cell reconstitution, and improves host defense against infections (18). In human clinical trials, IL-7 treatment has successfully restored lymphocyte counts, particularly T cells, in septic shock (19), HIV infection (20, 21), and severe COVID-19 (22). These studies consistently report expanded peripheral T cell populations and improved T cell immunity.

Importantly, native IL-7 has a short half-life, limiting its clinical application (23). To address this limitation, a novel long-acting recombinant human IL-7 (rhIL-7-hyFc) was developed by linking IL-7 to a hybrid IgD/IgG4 Fc domain, which increased its half-life by 5.4-fold (24). Previous studies of rhIL-7-hyFc have shown promising results. In murine tumor models, rhIL-7-hyFc increased peripheral lymphocyte counts and induced tumor regression (25–27). In early-phase trials involving healthy individuals, a single administration elicited robust and durable expansions of circulating T cells across naive and memory compartments (28, 29). Functional profiling demonstrated that expanded T cells maintained effector functionality and T cell receptor (TCR) repertoire diversity, without expansion of regulatory T (Treg) cells (28). More recently, a phase Ib study of patients with locally advanced or metastatic solid tumors (ClinicalTrials.gov NCT03478995) reported that rhIL-7-hyFc administration induced a marked increase in the number of circulating CD8^+^ and CD4^+^ T cells and the frequency of proliferating cells (30). Tumor biopsies obtained before and after treatment showed a significant increase in the number of tumor-infiltrating lymphocytes.

However, previous studies did not examine distinct transcriptomic features of peripheral blood T cells in response to rhIL-7-hyFc treatment and molecular mechanisms. In the present study, we performed single-cell RNA sequencing (scRNA-seq) and multicolor flow cytometry to investigate the effects of rhIL-7-hyFc on peripheral blood T cells in patients with locally advanced or metastatic solid tumors. Transcriptomic analysis revealed IL-7–induced pathways linked to T cell activation, survival, and proliferation. Repeated rhIL-7-hyFc administration was less effective; thus, we further investigated molecular features underlying IL-7 signaling hyporesponsiveness.

## Results

### Single-cell transcriptomes of peripheral blood T cells from patients with solid cancer treated with rhIL-7-hyFc.

**Figure 1:**
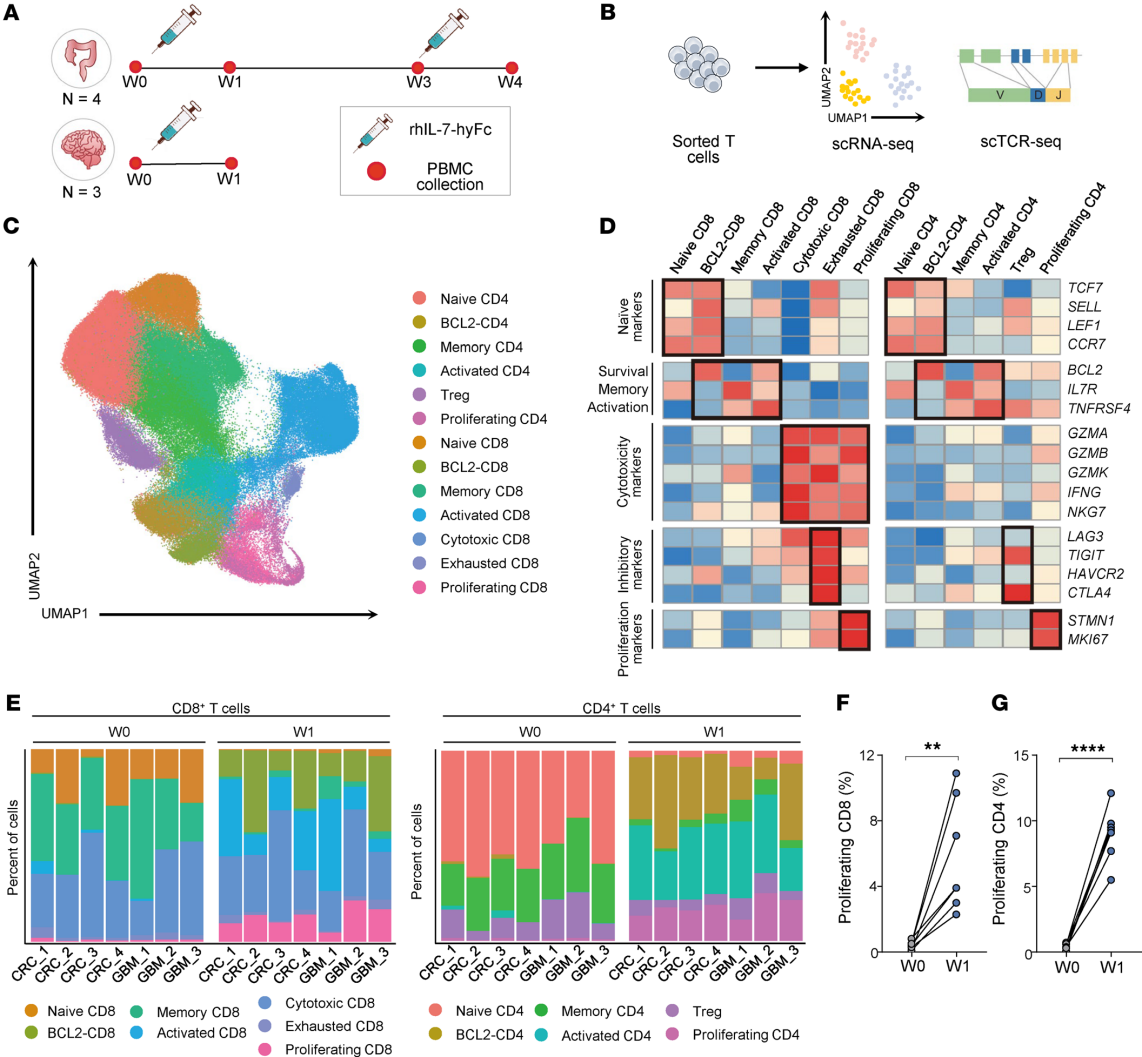
Study design. (**A**) Schematic overview of sample collection. PBMCs were collected from patients with colorectal cancer (CRC; *n* = 4) and glioblastoma (GBM; *n* = 3) before and after rhIL-7-hyFc administration. CRC patients received 2 doses. (**B**) Schematic illustration of the single-cell sequencing workflow. To assess the effects of rhIL-7-hyFc on peripheral T cells, sorted T cells were subjected to scRNA-seq and scTCR-seq. (**C**) Cell-type annotation of single cells. A total of 185,331 cells were first annotated as CD4^+^ or CD8^+^ T cells, and then further classified using canonical gene markers. (**D**) Expression of canonical markers in T cells. The expressions of well-established genes associated with a naive state, survival, memory, activation, cytotoxicity, inhibition, and proliferation were plotted to characterize each cluster. (**E**) Proportions of the CD8^+^ T cell (left) and CD4^+^ T cell (right) subsets per patient at baseline (W0) and 1 week after treatment (W1). (**F** and **G**) Proportion of proliferating CD8 (**F**) and CD4 (**G**) cluster per sample, based on scRNA-seq data from W0 and W1 (*n* = 7). Paired *t* test was used for comparison. **P < 0.01, ****P < 0.0001.

We performed scRNA-seq and multicolor flow cytometry using available peripheral blood mononuclear cells (PBMCs) obtained from 38 participants enrolled in recent phase Ib clinical trials evaluating the efficacy of rhIL-7-hyFc in patients with locally advanced or metastatic solid tumors (NCT03478995) or glioblastoma (GBM) (NCT03619239). The demographic and clinical characteristics of these 38 patients are presented in Supplemental Table 1; supplemental material available online with this article; https://doi.org/10.1172/jci.insight.203629DS1 Among them, PBMCs from 7 patients — 4 with colorectal cancer (CRC) and 3 with GBM — were used for scRNA-seq to comprehensively investigate the effects of rhIL-7-hyFc on peripheral blood T cells. PBMCs obtained at baseline (W0) and 1 week after treatment (W1) from all 7 patients were used. In the 4 patients with CRC, PBMCs obtained just before (W3) and 1 week after (W4) the second dose administration were also used (Figure 1A).

From PBMCs, T cells were isolated using a pan T cell isolation kit and were analyzed by scRNA-seq and single-cell TCR sequencing (scTCR-seq) (Figure 1B). These analyses yielded transcriptomic profiles for 185,331 cells across 7 patients and 4 time points and showed 13 distinct clusters (Figure 1C). Principal component analysis revealed minimal diversity among patients having different cancer types (Supplemental Figure 1, A and B). W1 was segregated from the other time points (Supplemental Figure 1C). Minimal batch effects were confirmed (Supplemental Figure 2). T cells were classified into CD4^+^ and CD8^+^ subsets, and further divided based on canonical markers, including *CCR7*, *BCL2*, *IL7R*, *TNFRSF4*, *GZMB*, *HAVCR2*, *MKI67*, and *FOXP3* (Supplemental Figure 3A). CD8^+^ T cells formed 7 clusters: naive CD8, BCL2^hi^-CD8, memory CD8, activated CD8, cytotoxic CD8, exhausted CD8, and proliferating CD8. CD4^+^ T cells formed 6 clusters: naive CD4, BCL2^–^CD4 (BCL2^hi^-CD4), memory CD4, activated CD4, CD4 Treg, and proliferating CD4. These clusters were defined based on gene expression patterns including markers of a naive state (*TCF7*, *SELL*, *LEF1*, and *CCR7*), survival (*BCL2*), memory (*IL7R*), activation (*TNFRSF4*), cytotoxicity and effector function (*GZMA*, *GZMB*, *GZMK*, *IFNG*, and *NKG7*), inhibition (*LAG3*, *TIGIT*, *HAVCR2*, and *CTLA4*), and proliferation (*STMN1* and *MKI67*) (Figure 1D).

To focus on the effects of the first rhIL-7-hyFc dose, we assessed T cell composition at W0 and W1. The T cell profiles exhibited significant shifts after rhIL-7-hyFc administration. Several clusters were markedly expanded at W1, including BCL2^hi^-CD8, activated CD8, cytotoxic CD8, proliferating CD8, BCL2^hi^-CD4, activated CD4, and proliferating CD4 (Figure 1E and Supplemental Figure 3B). In consensus with previous findings (28), sample-wise comparison of the proportion of proliferating or CD4^+^ T cells revealed a dramatic increase, 0.39% at baseline to 5.82% after treatment for CD8^+^ T cells and 0.39% to 9.0% for CD4^+^ T cells (Figure 1, F and G). The notable changes in cluster proportion represent rhIL-7-hyFc–induced changes in peripheral blood T cell dynamics.

### Cell cycling and antiapoptotic features of peripheral blood T cells after rhIL-7-hyFc treatment.

**Figure 2:**
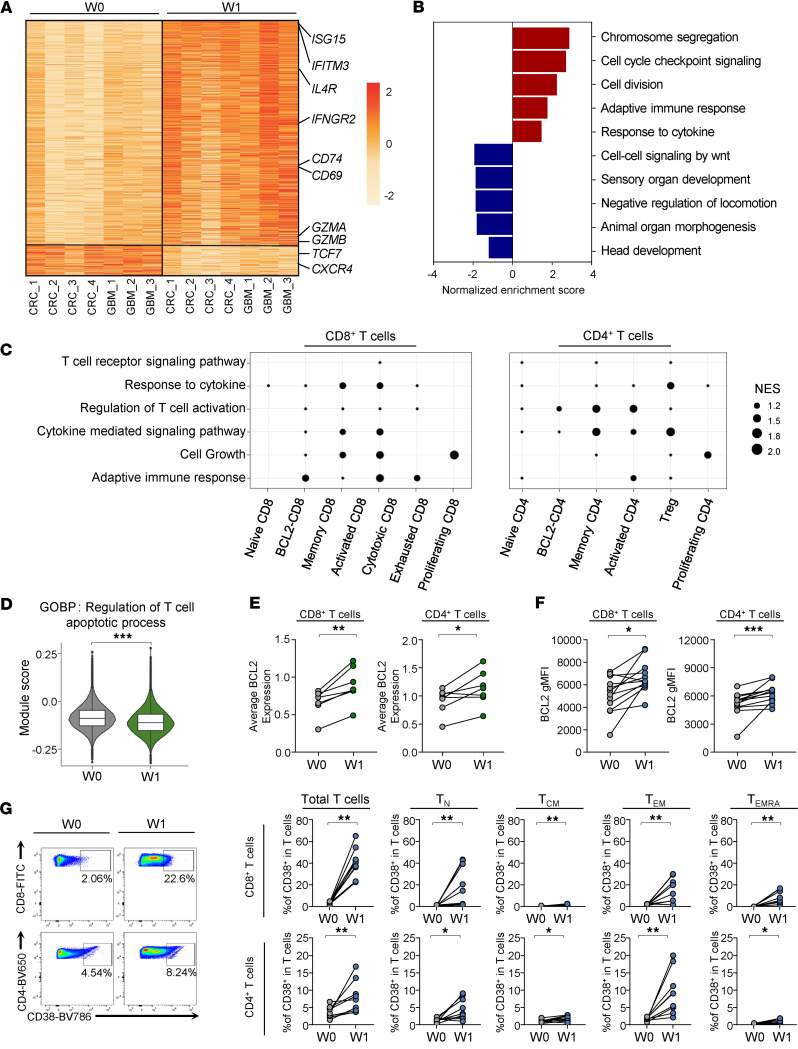
rhIL-7-hyFc–induced transcriptional changes in T cells. (**A**) Row-normalized heatmap of differentially expressed genes (DEGs) between W0 and W1. Representative genes are annotated. (**B**) Pathway enrichment analysis of DEGs. Pathways enriched at baseline (blue) and after rhIL-7-hyFc treatment (red) are shown. (**C**) GSEA of upregulated pathways after rhIL-7-hyFc treatment, among CD8^+^ and CD4^+^ T cells. (**D**) Module scores for the GO term “Regulation of T cell apoptotic process” were calculated and compared between W0 and W1. Unpaired *t* test was used for *P* value calculation. (**E** and **F**) Mean BCL2 expression per sample among CD8^+^ T cells and CD4^+^ T cells from scRNA-seq data (**E**) (*n* = 7) and from geometric mean fluorescence intensity (gMFI) measured by flow cytometry (**F**) (*n* = 12). (**G**) Flow cytometry–measured CD38 gMFI among CD8^+^ and CD4^+^ T cells, subdivided into total, naive, central memory, effector memory, and EMRA subsets (*n* = 9). Statistical testing was conducted using the paired Wilcoxon test (**E**–**G**). *P < 0.05, **P < 0.01, ***P < 0.001.

To examine rhIL-7-hyFc–induced features across T cell clusters, we performed differentially expressed gene (DEG) analysis between whole T cell transcriptomes from W0 and W1. This revealed 3,071 upregulated genes (notably including *CORO1B*, *ISG15*, *IFITM3*, *PSMB*, *IL4R*, *CD55*, *ISG20*, *CD5*, *CD7*, *IFNGR2*, *CD74*, *CD69*, *CEACAM1*, *IFITM1*, *IL32*, *ICOS*, *GZMA*, and *GZMB*) and 405 downregulated genes (notably including *DUSP1*, *BTG2*, *BTG1*, *IL7R*, *CXCR4*, *BCL11B*, *TCF7*, *DUSP2*, *CCL3*, *IL5RA*, and *IKZF2*) after treatment (Figure 2A).

Gene ontology (GO) analysis of the upregulated genes revealed enrichment of cell cycle and cell division processes, which is consistent with the expected effects of IL-7 treatment (Figure 2B, red bars). On the other hand, downregulated genes were associated with developmental processes not related to T cell function, such as organ development and morphogenesis (Figure 2B, blue bars). These findings were supported by gene set enrichment analysis (GSEA), which revealed significant enrichment of lymphocyte activation and cell growth after rhIL-7-hyFc treatment (Figure 2C and Supplemental Figure 4, A and B).

We also analyzed the expression of cytotoxic genes, including *GZMA*, *GZMB*, and *GZMK*, on a per-cell basis within each cluster. While the cytotoxic CD8 cluster proportion did not markedly increase (Supplemental Figure 3B), per-cell expression of cytotoxic genes within this cluster was substantially higher at W1 compared to W0 (Supplemental Figure 4C). We further examined granzyme genes across all CD8^+^ T cells, per cluster, and per patient at W0 versus W1 (Supplemental Figure 4C). This analysis confirms that cytotoxic gene expression increased at W1 both at the bulk CD8^+^ level and within individual clusters, particularly the cytotoxic CD8 and exhausted CD8 subsets, and that this pattern was consistent across patients.

Due to the increased proportions of the BCL2^hi^-CD8 and BCL2^hi^-CD4 clusters after rhIL-7-hyFc treatment, we next examined apoptosis-related genes. We calculated module scores for genes involved in regulating T cell apoptosis, curated by GO biological process (GOBP). These module scores were significantly lower at W1 compared with W0, indicating reduced apoptotic features after rhIL-7-hyFc treatment (Figure 2D). Moreover, *BCL2* mRNA levels were significantly higher at W1 across the CD8^+^ and CD4^+^ T cell clusters (Figure 2E). To validate BCL2 expression at the protein level, we performed flow cytometry analysis on PBMCs from an expanded cohort (*n* = 29), including 7 patients whose T cells had been analyzed by scRNA-seq. Flow cytometry revealed significantly increased BCL2 protein expression in total CD8^+^ and CD4^+^ T cells after rhIL-7-hyFc treatment (Figure 2F). In subset analysis, BCL2 expression was significantly increased in the naive subset (T_N_) and in all 4 subsets of CD8^+^ and CD4^+^ T cells (Supplemental Figure 4D). Thus, both single-cell and flow cytometry analyses revealed that rhIL-7-hyFc induced qualitative changes in T cells, including upregulation of cell cycle, activation of immune response, and induction of antiapoptosis.

Moreover, T cells treated with rhIL-7-hyFc exhibited increased activation, as evidenced by the increased populations of OX40^+^CD4^+^ and OX40^+^CD8^+^ T cells. Flow cytometry analysis (*n* = 9) also confirmed the upregulation of activation markers, such as CD38, HLA-DR, and OX40, particularly in the effector memory T (T_EM_) and CD45RA^+^ effector memory (T_EMRA_) cell populations (Figure 2G and Supplemental Figure 4, E and F).

### Temporal determination of T cell state in response to rhIL-7-hyFc.

**Figure 3:**
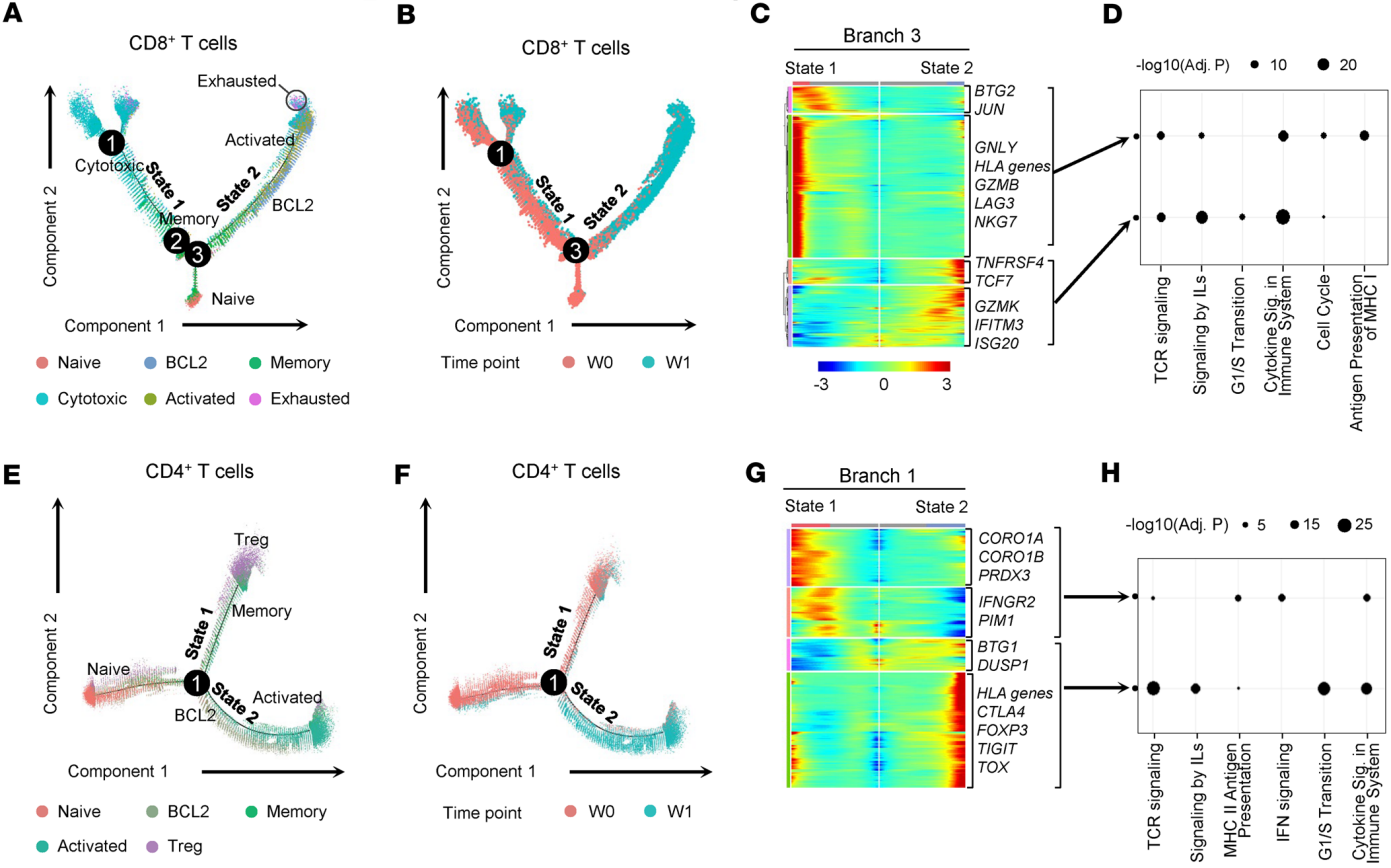
Pseudotime analysis of T cells after rhIL-7-hyFc treatment. (**A** and **B**) Pseudotime trajectory of CD8^+^ T cells was inferred using Monocle2. Cell annotation based on transcriptomic profile (**A**) and sampling time points (**B**) was mapped. Branch 3 divided state 1 versus 2. (**C** and **D**) Differentially expressed genes (DEGs) contributing to state bifurcation at branch 3 were identified by branch expression analysis modeling (BEAM) (**C**), and subjected to pathway enrichment analysis (**D**). Genes contributing to each state are marked with arrow. (**E** and **F**) Pseudotime trajectory of CD4^+^ T cells was inferred. Cell annotation based on transcriptomic profile (**E**) and sampling time points (**F**) was mapped. (**G** and **H**) DEGs contributing to CD4^+^ T cell state bifurcation were identified by BEAM (**G**), and analyzed by pathway enrichment (**H**).

We performed Monocle2 analysis to better understand dynamic changes of CD8^+^ and CD4^+^ T cell state induced by rhIL-7-hyFc. Proliferating T cell populations were excluded from trajectory construction. The trajectory of CD8^+^ T cells generated 3 branches, with the naive CD8^+^ T cell cluster as the starting point (Supplemental Figure 5A). Two prominent branches were observed at branch point 3 — with the left branch (state 1) representing the progression of memory CD8^+^ T cells into cytotoxic CD8^+^ T cells, and the right branch (state 2) illustrating the trajectory of BCL2^hi^-CD8^+^ into activated CD8^+^ T cells (Figure 3A). The state 2 branch ended at exhausted CD8^+^ T cells, although their relative frequency was negligible. Notably, state 1 was enriched with cells from time point W0, and state 2 was enriched with cells from W1 (Figure 3B).

To further investigate genes involved in the state decisions, we conducted branch expression analysis modeling (BEAM) analysis (Figure 3C). Pathway analysis of significant genes revealed that state 1 — mainly comprising CD8^+^ T cells from W0 — was enriched for TCR signaling and antigen presentation of MHC I (Figure 3D). State 2 — mainly comprising CD8^+^ T cells from W1 — was significantly enriched with terms including signaling by interleukins and cytokine signaling in immune system, reflecting the direct effects of rhIL-7-hyFc treatment. Additionally, state 2 exclusively showed upregulation of G_1_/S transition, suggesting increased cell proliferation.

Among CD4^+^ T cells, the trajectory construction yielded 2 paths from branch point 1, with pseudotime starting from the naive CD4^+^ T cell population (Supplemental Figure 5B). The upper branch (state 1) shows progression into memory CD4^+^ T cells and CD4^+^ Treg cells, while the lower arm (state 2) shows the trajectory of BCL2^hi^-CD4^+^ T into activated CD4^+^ T cells (Figure 3E). The 2 CD4^+^ T cell states were also differentially enriched across time points (Figure 3F).

We performed BEAM analysis to identify significant genes related to state decisions (Figure 3G). State 1 — mainly comprising CD4^+^ T cells from W0 — showed weaker enrichment of TCR signaling and cytokine signaling in the immune system, but stronger enrichment of MHC II antigen presentation and interferon signaling (Figure 3H). On the other hand, state 2 — mainly comprising CD4^+^ T cells from W1 — was highly enriched with terms representing cytokine and TCR signaling, as well as G_1_/S transition, indicating rhIL-7-hyFc–induced effects (Figure 3H). Thus, the trajectory analyses of CD8^+^ and CD4^+^ T cells revealed differential temporal enrichment patterns across cell states, characterized by strong enrichment of cytokine signaling pathways and G_1_/S phase transition, highlighting the immunomodulatory effects of rhIL-7-hyFc on peripheral blood T cells.

### Hyporesponsiveness after repeated rhIL-7-hyFc treatment.

**Figure 4:**
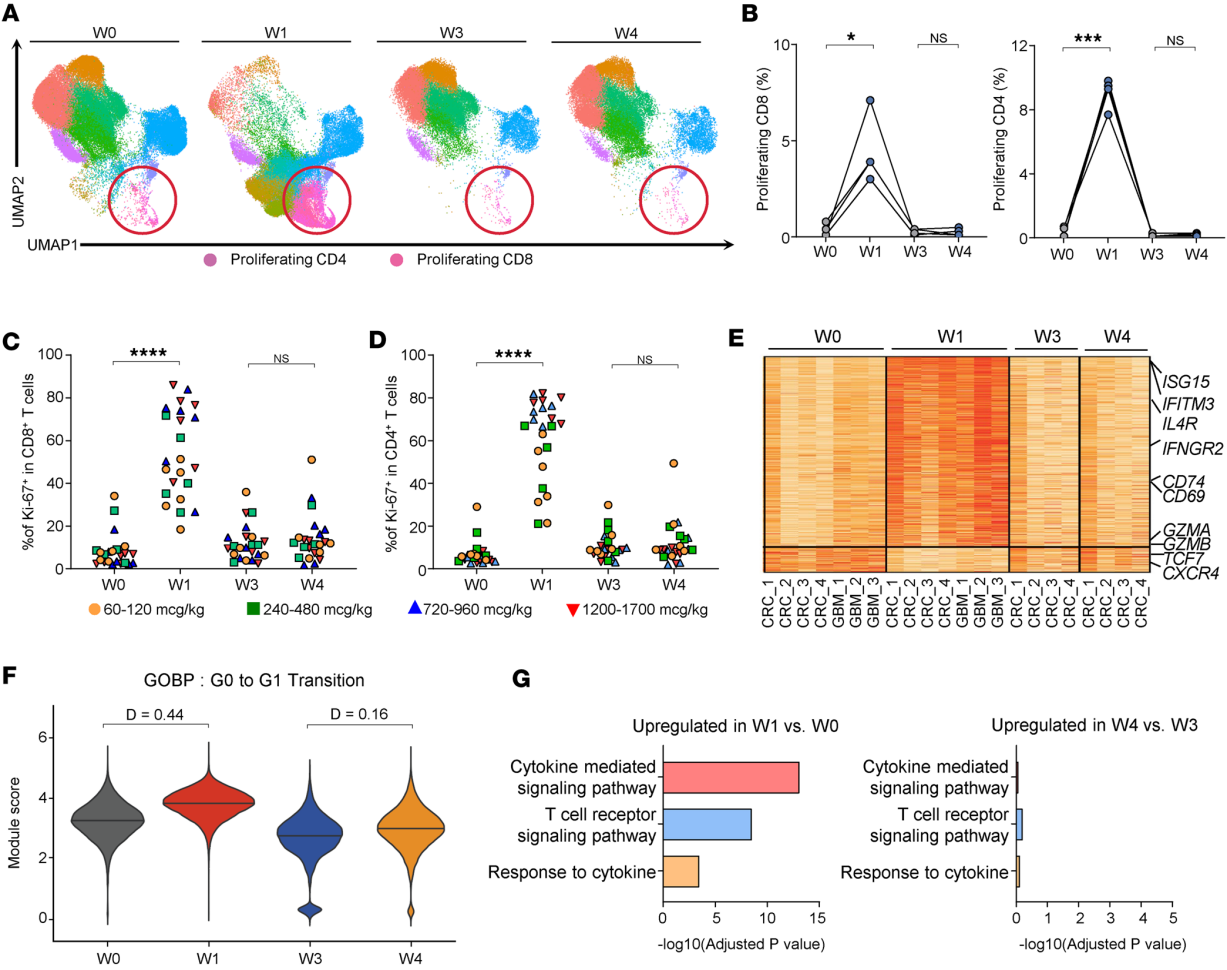
Hyporesponsiveness of T cells after repeated rhIL-7-hyFc administration. (**A**) UMAP of T cells from each time point, with proliferating T cells highlighted in red. (**B** and **C**) The frequency of proliferating CD8 (**B**) and CD4 (**C**) clusters at each time point was quantified using scRNA-seq data. Paired *t* tests were used for statistical comparison. (**D**) The proportions of Ki-67^+^ CD8^+^ (left) and CD4^+^ (right) T cells among peripheral T cells were measured across time points at different doses: 60–120 μg/kg (*n* = 6), 240–480 μg/kg (*n* = 5), 720–960 μg/kg (*n* = 6), and 1200–1700 μg/kg (*n* = 6). (**E**) Differentially expressed genes (DEGs) from W0 to W1 were projected across all time points using heatmap. (**F**) Comparison of module scores for the GO biological process “G_0_-to-G_1_ transition” between time points. Statistical differences were assessed using the Kolmogorov-Smirnov *D* value. (**G**) Comparison of immune activation–related pathway enrichment between the first and second rhIL-7-hyFc doses. *P < 0.05; ***P < 0.001; ****P < 0.0001.

In a recent phase Ib clinical trial (NCT03478995), it was reported that the second rhIL-7-hyFc administration at W3 failed to elicit a significant increase in T cell numbers (30). We investigated molecular mechanisms underlying this hyporesponsiveness. Consistent with the previous finding, scRNA-seq revealed distinct transcriptomic changes following the first administration (W1), while the second dose did not induce comparable transcriptomic changes (W4) (Figure 4A). The proportions of proliferating CD8^+^ and CD4^+^ T cell clusters were not increased after the second dose administration (Figure 4, B). This finding was further confirmed by flow cytometry analysis using PBMCs (*n* = 23), which revealed that the frequency of Ki-67^+^ T cells significantly increased at W1 compared with W0, but remained unchanged between W3 and W4 (Figure 4, C and D).

Treatment-induced transcriptomic profile changes were also distinct between the first and second administrations. The DEGs identified at W4 were not similar to the patterns identified at W1 (Figure 4E). Rather, DEG analysis between W3 and W4 revealed genes having little relevance to T cells or their functionality (Supplemental Table 2). In accordance with these findings, the G_0_-to-G_1_ transition term, which includes genes required for exiting quiescence and entering the cell division phase, showed an increased module score from W0 to W1 (*D* = 0.44), but only a marginally increased module score between W3 and W4 (*D* = 0.16), as measured by the Kolmogorov-Smirnov *D* statistic (Figure 4F). Additionally, expression of the key antiapoptotic gene *BCL2* was markedly increased at W1, but not at W4 (Supplemental Figure 6).

Finally, we compared the functional enrichment of upregulated genes across the 2 treatment periods. While W1 showed enrichment of T cell activation terms, such as response to cytokine or positive regulation of cytokine release, these terms were not enriched at W4 (Figure 4G). Overall, these findings indicate that repeated treatment failed to induce peripheral T cell proliferation or activation.

### Hyporesponsiveness is not attributed to anti-drug antibodies or receptor modulation.

**Figure 5:**
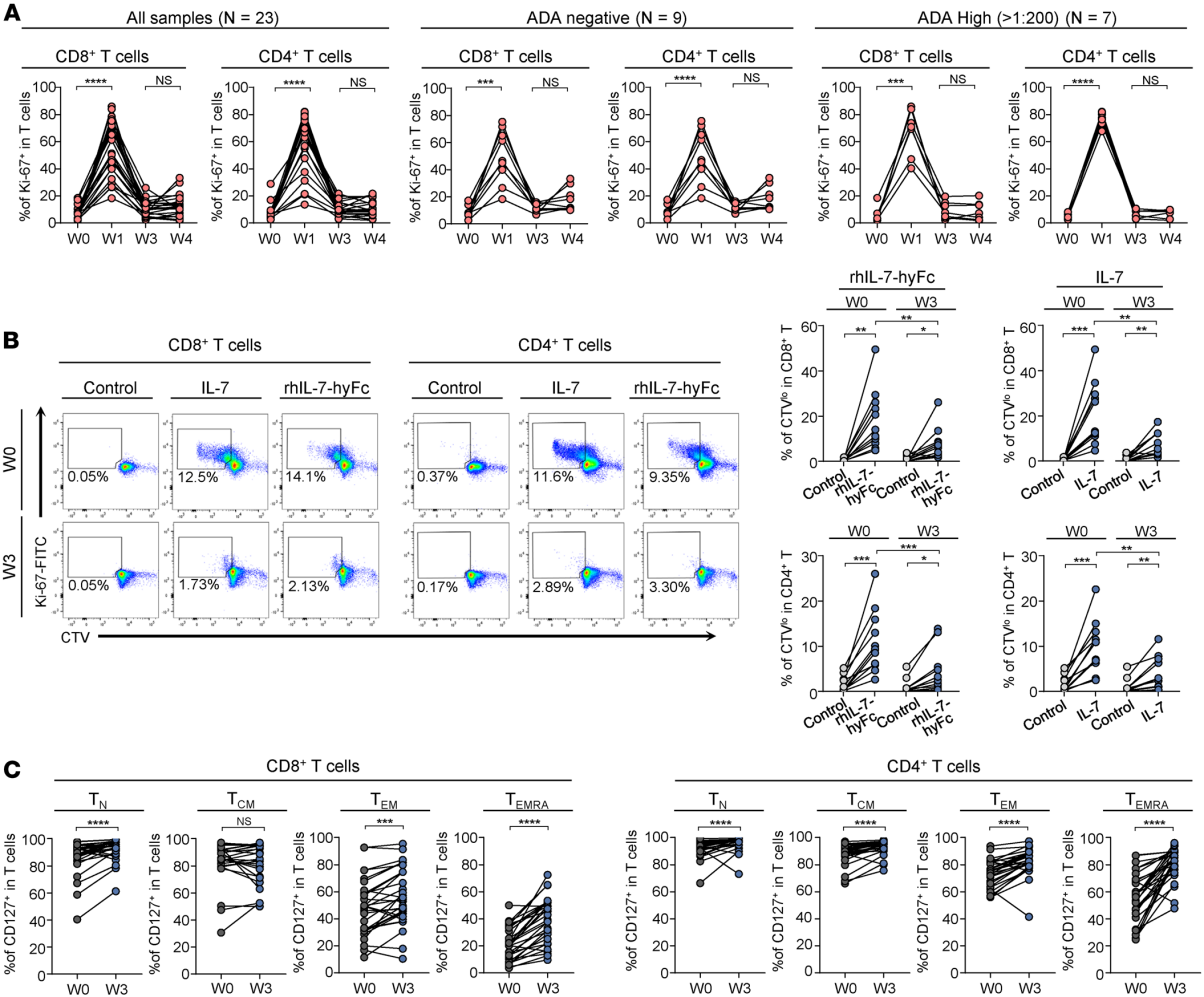
Impaired induction of T cell proliferation following repeated rhIL-7-hyFc administration. (**A**) Proliferative responses of CD8^+^ T cells (left) and CD4^+^ T cells (right) were measured in all samples (*n* = 23). The data were further divided into ADA-negative individuals (titer = 0; *n* = 9), and ADA-high individuals (>1:200; *n* = 7). (**B**) PBMCs collected at W0 and W3 (*n* = 11) were stained with CellTrace Violet (CTV), and stimulated for 120 hours with native IL-7 (10 ng/mL) or rhIL-7-hyFc (60 ng/mL). Representative flow cytometry plots for CD8^+^ T and CD4^+^ T cells (left), and the cumulative data regarding CTV^lo^ T cells after stimulation (right), are shown. (**C**) The frequency of CD127^+^ T cells within the CD8^+^ (top) and CD4^+^ (bottom) subsets were assessed for W0 and W3 (*n* = 29). Statistical analyses were conducted with the Wilcoxon test. NS, not significant. *P < 0.05; **P < 0.01; ***P < 0.001; ****P < 0.0001.

To investigate mechanisms underlying the hyporesponsiveness observed after the second rhIL-7-hyFc treatment, we examined various factors that could influence the effects of rhIL-7-hyFc. We first hypothesized that rhIL-7-hyFc might elicit anti-drug antibodies (ADAs) that could neutralize the effect of the second dose. Therefore, we measured the ADA titers of serum samples obtained after the first administration (W3). Among 23 individuals, 14 were ADA-positive and 9 were ADA-negative. To further examine the effect of ADAs on responsiveness to rhIL-7-hyFc, we compared the frequency of Ki-67^+^ cells among CD8^+^ and CD4^+^ T cells between the ADA-negative group (*n* = 9) and individuals with a high ADA titer (>1:200) (*n* = 7). However, both patient groups showed a significantly decreased Ki-67^+^ frequency at W4 compared with W1 (Figure 5A), indicating that ADAs cannot fully explain the hyporesponsiveness observed upon the second administration.

Next, we treated PBMCs obtained at W0 and W3 with rhIL-7-hyFc or rhIL-7 ex vivo, and examined the frequency of CellTrace Violet–low (CTV^lo^) proliferating cells among CD8^+^ and CD4^+^ T cells. In this experiment, we could exclude any potential effect of ADAs, because the culture did not include autologous sera. We found that rhIL-7-hyFc treatment significantly increased the CTV^lo^ cell frequency among CD8^+^ and CD4^+^ T cells obtained at both W0 and W3. However, the rhIL-7-hyFc–induced increase in the CTV^lo^ frequency was significantly lower among CD8^+^ and CD4^+^ T cells obtained at W3 compared with those obtained at W0 (Figure 5B). Similar results were observed when PBMCs were treated with rhIL-7. These findings corroborate that the hyporesponsiveness is attributed to cell-intrinsic factors, and not ADAs.

We also hypothesized that the expression level of IL-7Rα (CD127) on the T cell membrane at W3 might be downregulated compared with W0. However, IL-7Rα expression was instead upregulated in all subsets of CD8^+^ T cells at W3 compared with W0, with the exception of CD8^+^ T_CM_ cells (Figure 5C). All 4 subsets of CD4^+^ T cells exhibited the same result. These findings indicate that the observed hyporesponsiveness is not attributable to the downregulation of IL-7Rα.

### Intracellular disruption of distal signaling accounts for IL-7 hyporesponsiveness.

**Figure 6:**
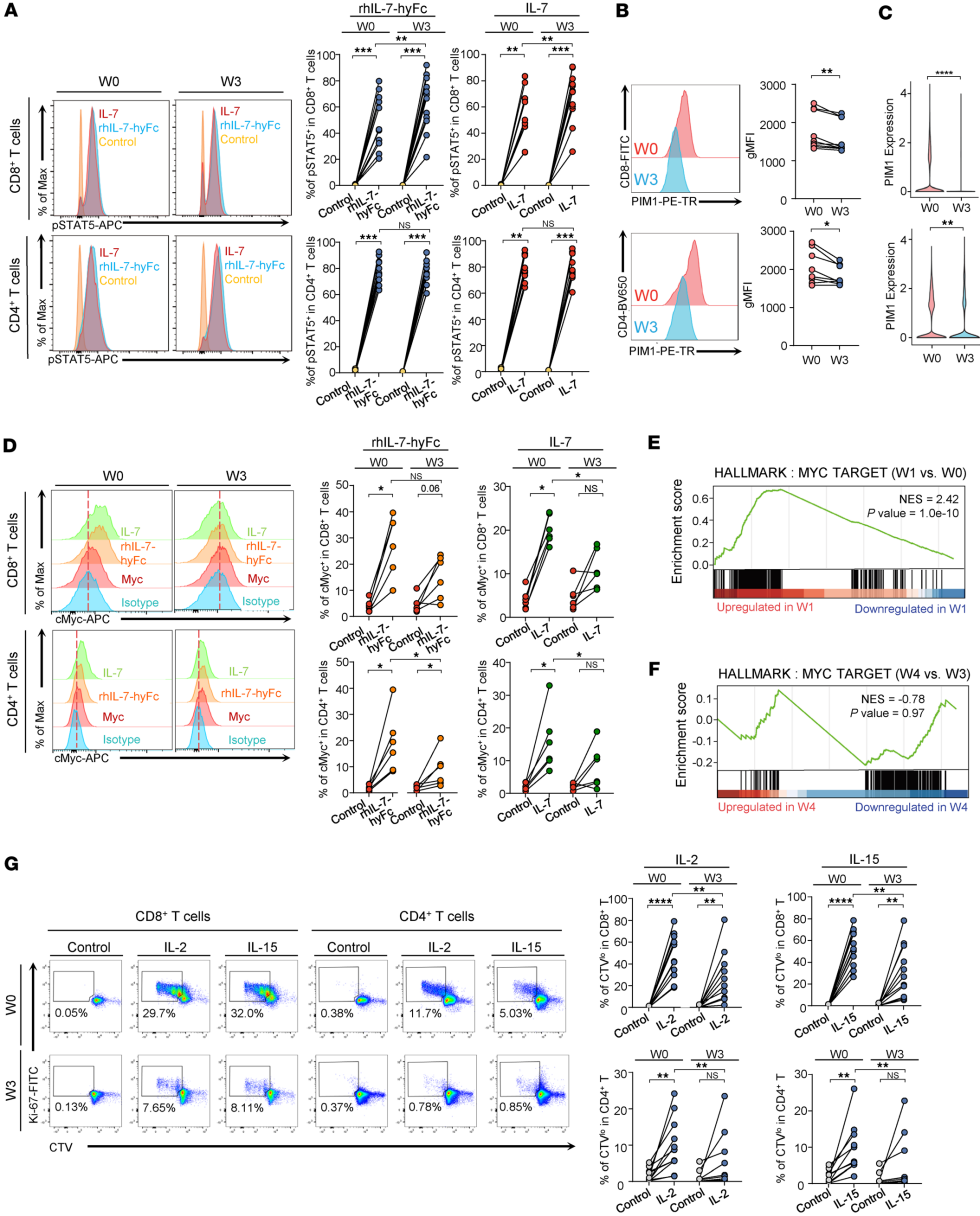
Altered cytokine signaling following repeated rhIL-7-hyFc treatment. (**A**) PBMCs collected at W0 and W3 were incubated for 120 hours with no stimulation or with stimulation using native IL-7 (10 ng/mL) or rhIL-7-hyFc (60 ng/mL). Subsequently, the frequency of p-STAT5^+^ cells among T cells was measured (*n* = 11 for rhIL-7-hyFc and *n* = 10 for IL-7). (**B**) PIM-1 kinase expression was assessed by gMFI in CD8^+^ (upper) and CD4^+^ (lower) T cells from W0 and W3 (*n* = 9). (**C**) *PIM1* expression at the single-cell resolution is plotted for CD4^+^ (upper) and CD8^+^ (lower) T cells. (**D**) Induction of c-Myc was assessed in CD8^+^ (upper) and CD4^+^ (lower) T cells from W0 and W3 (*n* = 6). (**E** and **F**) GSEA was performed to measure enrichment of HALLMARK: MYC_TARGETS after the first dose (**E**) and the second dose (**F**). (**G**) PBMCs from W0 and W3 (*n* = 8) were stimulated for 120 hours with rhIL-2 (100 IU/mL) or rhIL-15 (10 ng/mL). The cumulative data regarding CTV^lo^ T cell frequencies after stimulation are shown on the right. Statistical comparisons were performed using paired *t* tests. The control data for **G** are shared with Figure 5B, as both panels were derived from the same experiment designed to compare donor PBMC proliferative capacity following stimulation with rhIL-7 or rhIL-7-hyFc (Figure 5B) and rhIL-2 or rhIL-15 (**G**). NS, not significant. *P < 0.05; **P < 0.01; ***P < 0.001; ****P < 0.0001.

We next investigated whether the proximal signaling of IL-7Rα was attenuated in T cells obtained at W3 compared with those at W0. To this end, we treated PBMCs from W0 and W3 with rhIL-7-hyFc (*n* = 11) or rhIL-7 (*n* = 10), and examined the level of phosphorylated STAT5 (p-STAT5). Treatment with rhIL-7-hyFc or rhIL-7 significantly increased the p-STAT5 level in CD8^+^ T cells obtained at both W0 and W3 (Figure 6A). Importantly, the rhIL-7-hyFc–induced increase in p-STAT5 did not differ between CD8^+^ T cells obtained at W0 versus W3, and similar results were observed in CD4^+^ T cells. These data indicate that the proximal signaling of IL-7Rα was not disrupted in T cells obtained at W3.

Next, we examined PIM-1 kinase, a downstream target of p-STAT5 (31) which is involved in cell cycle regulation. We performed flow cytometry to measure PIM-1 expression in CD8^+^ and CD4^+^ T cells obtained at W0 and W3 (*n* = 9), and found significantly lower PIM-1 levels at W3 compared with W0 (Figure 6B). This reduction in *PIM1* expression was additionally confirmed by scRNA-seq data (Figure 6C). This low PIM-1 kinase expression at W3 may contribute to the IL-7 hyporesponsiveness.

We also analyzed the expression of STAT5 downstream target genes across T cell clusters in scRNA-seq data. We computed a STAT5 transcriptional activity score using the MSigDB HALLMARK_IL2_STAT5_SIGNALING gene set as a proxy for STAT5 downstream output. The STAT5 target gene module score was elevated at W1 compared with W0 across most clusters (Supplemental Figure 7A). In contrast, comparing W4 to W3, the STAT5 target gene score showed minimal change, indicating attenuated STAT5 transcriptional output following the second dose. *PIM1* expression also showed a concordant pattern across T cell clusters (Supplemental Figure 7B).

We additionally examined the expression of c-Myc, a critical regulator of cell proliferation, which is downstream of PIM-1 (32, 33). PBMCs obtained at W0 and W3 were treated with rhIL-7-hyFc (*n* = 6) or rhIL-7 (*n* = 6) ex vivo. Either treatment led to significantly increased c-Myc expression in CD8^+^ T cells obtained at W0, but not in CD8^+^ T cells obtained at W3 (Figure 6D). A similar trend was observed among CD4^+^ T cells, although rhIL-7-hyFc significantly increased the c-Myc expression level in CD4^+^ T cells obtained at W3. GSEA conducted on scRNA-seq data confirmed that Myc-targeted genes were upregulated after first dose, but not after the second dose (Figure 6, E and F).

Our results indicated that the distal signaling of IL-7Rα, at the stage of PIM-1 kinase/c-Myc, was disrupted in T cells obtained at W3. Thus, we hypothesized that these T cells might also exhibit hyporesponsiveness to other common γ-chain cytokines that share the same distal signaling of PIM-1/c-Myc. To investigate this possibility, we treated PBMCs obtained at W0 and W3 with rhIL-2 or rhIL-15. Our results showed that rhIL-2 or rhIL-15 treatment significantly increased the CTV^lo^ cell frequency among CD8^+^ T cells obtained at both W0 and W3 (Figure 6G). However, the rhIL-2– or rhIL-15–induced increase in the CTV^lo^ frequency was significantly lower among CD8^+^ T cells obtained at W3 compared with those obtained at W0. Moreover, rhIL-2 or rhIL-15 treatment significantly increased the CTV^lo^ cell frequency among CD4^+^ T cells obtained at W0, but not among CD4^+^ T cells obtained at W3. These data confirmed that T cells obtained at W3 also exhibited partial hyporesponsiveness to other common γ-chain cytokines, such as IL-2 and IL-15, supporting the notion that disruption in the distal signaling of PIM-1/c-Myc accounts for the hyporesponsiveness observed after the second rhIL-7-hyFc administration.

## Discussion

In this study, we used single-cell transcriptomics and flow cytometry to investigate the effects of the long-acting rhIL-7-hyFc on peripheral T cells from patients with locally advanced or metastatic solid tumors. Administration of rhIL-7-hyFc resulted in marked expansion of Ki-67^+^ T cells, and induced transcriptional changes associated with cell cycle progression, immune activation, and antiapoptosis. Trajectory inference further revealed that W1 T cells diverged from W0 cells along a distinct pseudotime branch, and that this separation of trajectories was partially driven by cytokine and TCR signaling pathways. These findings suggest that rhIL-7-hyFc can drive cell proliferation and a transcriptional shift.

Immune checkpoint blockade (ICB) therapy has transformed cancer therapy, but yields a durable clinical benefit in only a subset of patients (34–38). Importantly, lymphopenia is associated with poor outcomes following ICB, and absolute lymphocyte count has been proposed as a biomarker to predict therapeutic efficacy (4, 39, 40). Given its ability to expand CD4^+^ and CD8^+^ T cells without increasing exhaustion-associated markers, rhIL-7-hyFc may be useful as an adjuvant of ICB therapy in patients with lymphopenic cancer. In a next step of clinical studies, tumor control should be assessed after rhIL-7-hyFc administration with a various dosing schedule. In addition, tumor-infiltrating lymphocytes should be investigated after rhIL-7-hyFc administration.

IL-7 is a homeostatic cytokine essential for T cell survival, expansion, and maintenance, and signals via IL-7Rα (CD127) and the common γ-chain (CD132). Unlike other γ-chain cytokines, IL-7 promotes T cell persistence without driving terminal differentiation or exhaustion, making it a promising candidate for combination immunotherapy. Previous clinical studies have demonstrated the safety of rhIL-7-hyFc and its efficacy in increasing T cell numbers in both healthy volunteers and cancer patients (28, 30). In the present study, we have extended those findings by showing that rhIL-7-hyFc induced coordinated transcriptional remodeling, thereby enhancing proliferation and survival pathways.

Notably, we observed attenuated responses following the second rhIL-7-hyFc administration. While the first dose induced robust T cell proliferation and activation, the second dose failed to reproduce these effects, at both the transcriptomic and protein levels. Hyporesponsiveness following repetitive cellular stimulation has been reported in the broader context of immune cell dysfunction. For example, monocytes repeatedly challenged with innate immune ligands develop diminished cytokine production and impaired inflammatory responses (41).

To understand the IL-7 hyporesponsiveness, we strengthened the mechanistic inference with a stepwise approach that excluded major extracellular/extrinsic explanations and mapped the signaling changes along the IL-7 pathway. Specifically, we excluded ADAs and IL-7Rα downmodulation from possible mechanisms. We demonstrated a cell-intrinsic reduction in responsiveness using ex vivo stimulation, showed preserved proximal signaling (p-STAT5), and demonstrated attenuation of distal signaling elements (PIM-1 and c-Myc) along with reduced functional proliferation in response to IL-2 and IL-15 as well as IL-7.

It has been known that PIM kinase activity is functionally required for IL-7–driven proliferative responses. Ribeiro et al. showed that pharmacological PIM inhibition with AZD1208 reversed IL-7–mediated cell cycle progression and significantly reduced IL-7–mediated proliferation without affecting IL-7–mediated cell survival or BCL2 expression (42). Moreover, CD8^+^ and CD4^+^ T cells obtained at W3 also showed reduced responses to IL-2 and IL-15, indicating broader dysfunction in γ-chain cytokine signaling. Further research is needed to determine whether it may be attributed to the long-acting formulation of rhIL-7, or the short interval between the first and second doses.

This study has several limitations. First, PBMCs were used instead of tumor-infiltrating lymphocytes, which may not fully recapitulate the intratumoral immune landscape. Second, we only evaluated a single time point after the second dose, limiting evaluation of longitudinal response. Third, this study focused on transcriptional profiling, and did not assess additional layers of regulation, such as epigenetics, proteomics, and metabolism. Fourth, effects of rhIL-7-hyFc could not be compared to those of an unmodified form of IL-7 because this clinical study focused on the effect of rhIL-7-hyFc only. Finally, our cohort size and tumor type diversity were limited; thus, there remains a need for further studies in larger and more diverse cohorts.

In summary, rhIL-7-hyFc induces proliferation and activation of peripheral T cells in patients with advanced solid tumors. These data support the further development of rhIL-7-hyFc as an immune-restorative agent, and suggest its potential as an adjuvant to ICB or other immunotherapies. To the best of our knowledge, this study provides the first single-cell transcriptomic characterization of human T cell responses upon rhIL-7-hyFc treatment. Future studies should explore the dosing interval, define mechanisms of signaling attenuation, and evaluate impact in combination with ICB.

## Methods

### Sex as a biological variable.

Our study examined male and female individuals, and sex was not considered as a key biological variable.

### Patient and sample collection.

Patients were recruited in an open-label, dose-escalation, phase Ib trial designed to evaluate the safety, tolerability, pharmacokinetics, and pharmacodynamics of rhIL-7-hyFc in patients refractory to standard therapy for locally advanced or metastatic solid tumors (NCT03478995) or GBM (NCT03619239). All patients were Asian and submitted written informed consent prior to any study-related procedures. PBMCs and plasma samples were collected.

### scRNA-seq.

PBMCs were isolated from fresh blood using Ficoll separation (GE Healthcare), and then cryopreserved in fetal bovine serum (FBS) with 10% DMSO, using liquid nitrogen. Cryopreserved PBMCs were thawed with DNase to inhibit cell aggregation. T cells were isolated using a pan T cell isolation kit (130-096-535, Miltenyi Biotec). scRNA-seq libraries were generated using the Chromium Single Cell 5′ Library & Gel Bead Kit, 5′ Feature Barcode Library Kit, and V(D)J Kit, Human T Cell, following the manufacturer’s instructions. Libraries were constructed, sequenced on the Illumina 10X platform, and sequencing read quality was evaluated using the Bioanalyzer (Agilent Technologies). The sequencing reads were aligned to the GRCh38 human reference genome using Cell Ranger version 3.1.0 (https://www.10xgenomics.com/support/software/cell-ranger/latest).

### Data preprocessing.

Using Seurat v4.0.5 (https://satijalab.org/seruat/), the following parameters were applied to ensure high-quality sequencing data: cells with less than 300 or more than 3000 unique transcripts were removed, cells with greater than 10% of transcripts mapped to mitochondrial genes were filtered, and genes detected in less than 3 cells were removed. Next, the data were normalized by using the NormalizeData function to log-normalize the total number of transcripts per cell to 10,000. Subsequently, the ScaleData function was applied while regressing on the patient identity to reduce variation between patients. Variable features were detected using FindVariableFeatures under default settings. To reduce dimensionality, the RunPCA function was performed on the scaled data using 50 components. To minimize batch effect, we used the RunHarmony function of the harmony R package (https://github.com/immunogenomics/harmony) to regress on patient. Next, the top 25 significant principle components were used to perform RunUMAP visualization. For accurate unsupervised clustering, the FindNeighbor function was applied using the first 25 principle components, as well as FindClusters using the Louvain algorithm with a resolution of 0.6.

Each cluster was first identified as CD4^+^ or CD8^+^ T cells based on expression of CD4, CD8A, and CD8B. Next, the differential gene expression per cluster was calculated using the FindAllMarkers function, with a logfc.threshold of greater than 1 to identify distinctive features. These results were used to conduct further classification using canonical markers of T cells, including *CCR7* (naive T cell marker), *BCL2* (antiapoptosis marker), *IL7R* (memory T cell marker), *TNFRSF4* (T cell activation marker), *FOXP3* (regulatory T cell marker), *GZMB* (cytotoxic T cell marker), *HAVCR2* (exhausted T cell marker), and *MKI67* (proliferating T cell marker). Canonical gene expression per cluster was visualized using the pheatmap R package. Furthermore, the cell cluster composition per sample was plotted with the dittoSeq R package.

### scRNA-seq data analysis.

Using the normalized and batch-corrected scRNA-seq data, DEG analysis was conducted with the FindMarkers function in Seurat. Significant DEGs were defined as those showing an adjusted *P* value of less than 1 × 10^–5^ and absolute log(fold change) of greater than 0.3. DEG heatmaps were plotted with the pheatmap R package. Pathway analysis was conducted using GSEA v4.3.2 in Windows. GOBP 2018 was used to check DEG enrichment in each pathway. Pathways with FDR *q* values of less than 0.25 were considered significantly enriched. Module scores of genes in a pathway were calculated using the AddModuleScore function of the Seurat package. These results were visualized using the ggplot2 R package. Calculation of a particular gene (namely, *BCL2*) in the scRNA-seq data of CD4^+^ or CD8^+^ T cells was performed using the AverageExpression function in the Seurat package. Since the data were previously log-normalized, no additional scaling was applied.

GSEA was performed using clusterProfiler with following gene sets: Lymphocyte activation involved in immune response, Cell growth from GOBP, and Myc targets v1 and v2 from HALLMARK. For analysis, the 2 Myc target pathways were combined.

Pseudotime trajectory analysis was conducted with Monocle v2.30.0 (https://cole-trapnell-lab.github.io/monocle-release/) using default parameters. We first selected a set of ordering genes, using DEGs between clusters. Next, reversed graph embedding was performed to reduce dimensionality, and single cells were projected and ordered into a trajectory with branch points. The trajectory starting point was set as a point containing naive CD4^+^ or CD8^+^ cells. BEAM was used to identify genes contributing to different cell states. Those having adjusted *P* values of less than 1 × 10^−150^ were considered significant genes. All analyses were conducted using default parameters, unless otherwise mentioned.

### Cell proliferation assay.

Cryopreserved PBMCs were stained with CTV (Thermo Fisher Scientific) according to the manufacturer’s instructions. Briefly, cells were incubated with CTV for 20 minutes at room temperature. Then, we added 1% FBS in PBS (Corning) to the cells to wash unbound dye. Cells were cultured in a 96-well plate at 0.5 million cells per well. Cells were stimulated with rhIL-2 (100 IU/mL), rhIL-7 (10 ng/mL), or rhIL-15 (10 ng/mL) from PeptroTech, or rhIL-7-hyFc, which was calculated at the same molarity as rhIL-7 considering its molecular weight (104 kDa) and used at 60 ng/mL. The cells were incubated for 120 hours, and then harvested into FACS tubes. Next, surface molecule staining and dead cell staining were performed for 15 minutes at room temperature using the following antibodies: anti-CD3-BV786 (clone UCHT1), anti-CD14-PE-TR (clone MφP9), anti-CD19-PE-TR (clone HIB19), anti-CD4-PE-Cy5 (clone RPA-T4), anti-CD8-AF700 (clone RTA-T8), and anti-CD45RA-APC-H7 (clone HI100) from BD Biosciences, and anti-CCR7-PerCP-Cy5.5 from BioLegend. Fixation and permeabilization were performed using the Foxp3/Transcription Factor Staining Buffer Set (Invitrogen), following the manufacturer’s instructions. Intracellular molecule staining with the antibody anti–Ki-67-FITC (clone B56) was performed for 15 minutes at room temperature, protected from light. Data were collected using a BD FACSymphony A3 instrument, and analyzed using FlowJo v10 software, BD Life Sciences).

### Flow cytometry analysis.

To identify the expression of cell surface markers, transcription factors, and phosphorylated proteins, FACS was performed using cryopreserved PBMCs that had been collected at specific time points. Surface molecules were stained for 15 minutes at room temperature with the following antibodies: anti-CD3-BV510 (clone UCHT1), anti-CD14-PE-TR (clone MφP9), anti-CD19-PE-TR (clone HIB19), anti-CD4-BV650 (clone SK3), and anti-CD8-FITC (clone RTA-T8) from BD Biosciences. Dead cells were stained using the Live/Dead Fixable Red Dead Cell Stain Kit (Thermo Fisher Scientific). Cells were fixed and permeabilized with IC Fixation Buffer (Thermo Fisher Scientific), and incubated at room temperature without light for 1 hour. After fixation, cells were washed with PBS, and then fixed with methanol (Sigma-Aldrich) for 1 hour on ice. Next, the cells were washed twice with excess PBS, and then stained for 30 minutes at room temperature using the following antibodies: anti–p-STAT5-APC (clone 47) from BD Biosciences, anti–c-Myc-APC (clone 9E10) from R&D Systems, and anti–PIM-1-AF594 (clone 12H8) from Santa Cruz Biotechnology.

### ADA test.

ADAs were determined by enzyme-linked immunosorbent assay (ELISA). Cryopreserved plasma samples were thawed and incubated in rhIL-7-hyFc–coated plates. Next, the plates were washed with PBS, and biotin-labeled rhIL-7-hyFc was added. Subsequently, the bound complex was detected using streptavidin–horseradish peroxidase (Strep-HRP). The addition of tetramethylbenzidine (TMB) substrate created colorimetric signal that was proportional to the formed antibody. Optical densities of formed complexes were measured at 450 nm.

### Statistics.

Statistical analysis was performed using GraphPad Prism version 9. The statistical difference between time points was estimated using the Wilcoxon signed-rank test (for 2 groups, paired) using 2-tailed *P* values. The differential distribution used for module score analysis was determined with the Kolmogorov-Smirnov *D* statistic. Differences were considered to be significant when *P* was less than 0.05.

### Study approval.

This study was approved by the institutional review board of each research site (Seoul St. Mary’s Hospital, Catholic University of Korea [KC18MDDF0139]; Asan Medical Center [2017-1215]; Samsung Medical Center [SMC 2018-04-041]; and Severance Hospital, Yonsei University College of Medicine [4-2018-0393]).

### Data availability.

The single-cell transcriptomics data generated from this study are available at Zenodo (https://zenodo.org/records/17958065). Values for all data points in graphs are reported in the Supporting Data Values file.

## Author contributions

HJ, JYK, SHP, and ECS designed the study. HK, MSB, MAL, JHC, DHN, TWK, SSJ, and JHS collected clinical samples and associated information. HJ, JYK, and SK performed the experiments and conducted data analysis and interpretation. HJ, JYK, SHP, and ECS wrote the manuscript with critical input from all authors. The order of the co–first authors was determined based on their respective roles and contributions to the study.

## Conflict of interest

HK and MSB are employees of Genexine, Inc.

## Funding support

- National Research Foundation of Korea (NRF) grants NRF-2022R1A2C3007292 and RS-2024-00439160 funded by the Korea government (MSIT).
- Ministry of Food and Drug Safety grant RS-2025-02213409.
- Korea Health Technology R&D Project grant RS-2025-25460003 through the Korea Health Industry Development Institute (KHIDI), funded by the Ministry of Health & Welfare, Republic of Korea.

## Supplementary Material

Supplemental data

ICMJE disclosure forms

Supplemental table 2

Supporting data values

## Version 1 04/21/2026

In-Press Preview

## Version 2 06/08/2026

Electronic publication
