# Supplementary material for: Long-acting IL-7 induces distinct transcriptomic features in peripheral T cells of patients with solid tumors
Source: JCI Insight. 2026 Apr 21;11(11):e203629. doi: 10.1172/jci.insight.203629 (PMC13313558; doi:10.1172/jci.insight.203629)
Supplement: Supplemental data [file jciinsight-11-203629-s220.pdf]

Supplementary data legends

Supplementary Figure 1

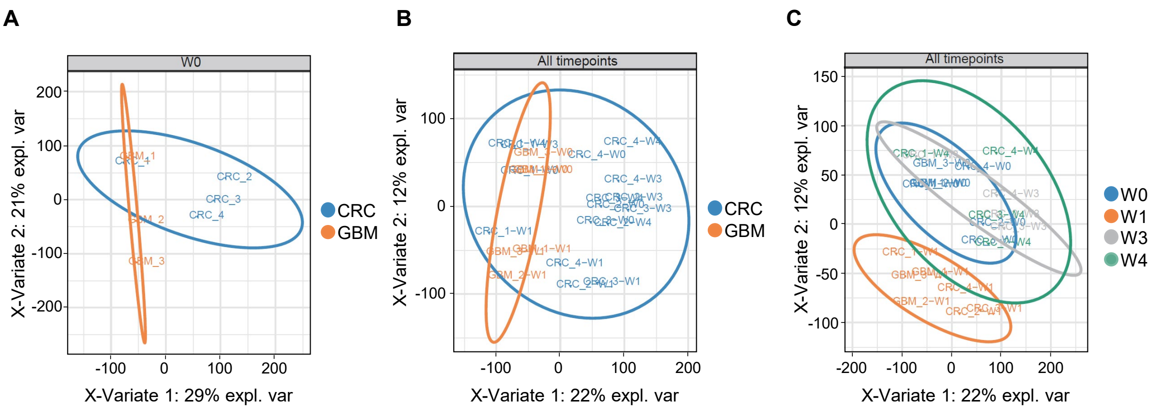

Supplementary Figure 1 Principal component analysis (PCA) of scRNA-seq. **(A)** PCA of baseline samples (W0) across patients. **(B-C)** PCA across time-points, grouped by cancer type (B), and by sampling time-point (C).

Supplementary Figure 2

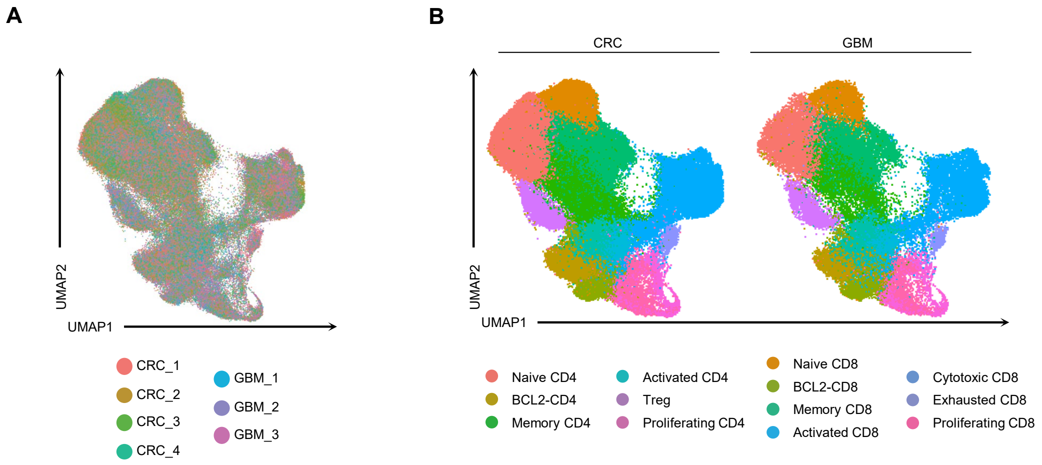

Supplementary Figure 2 Batch effects in the single-cell dataset. UMAP visualization grouped by patient (A), and cancer type (B).

Supplementary Figure 3

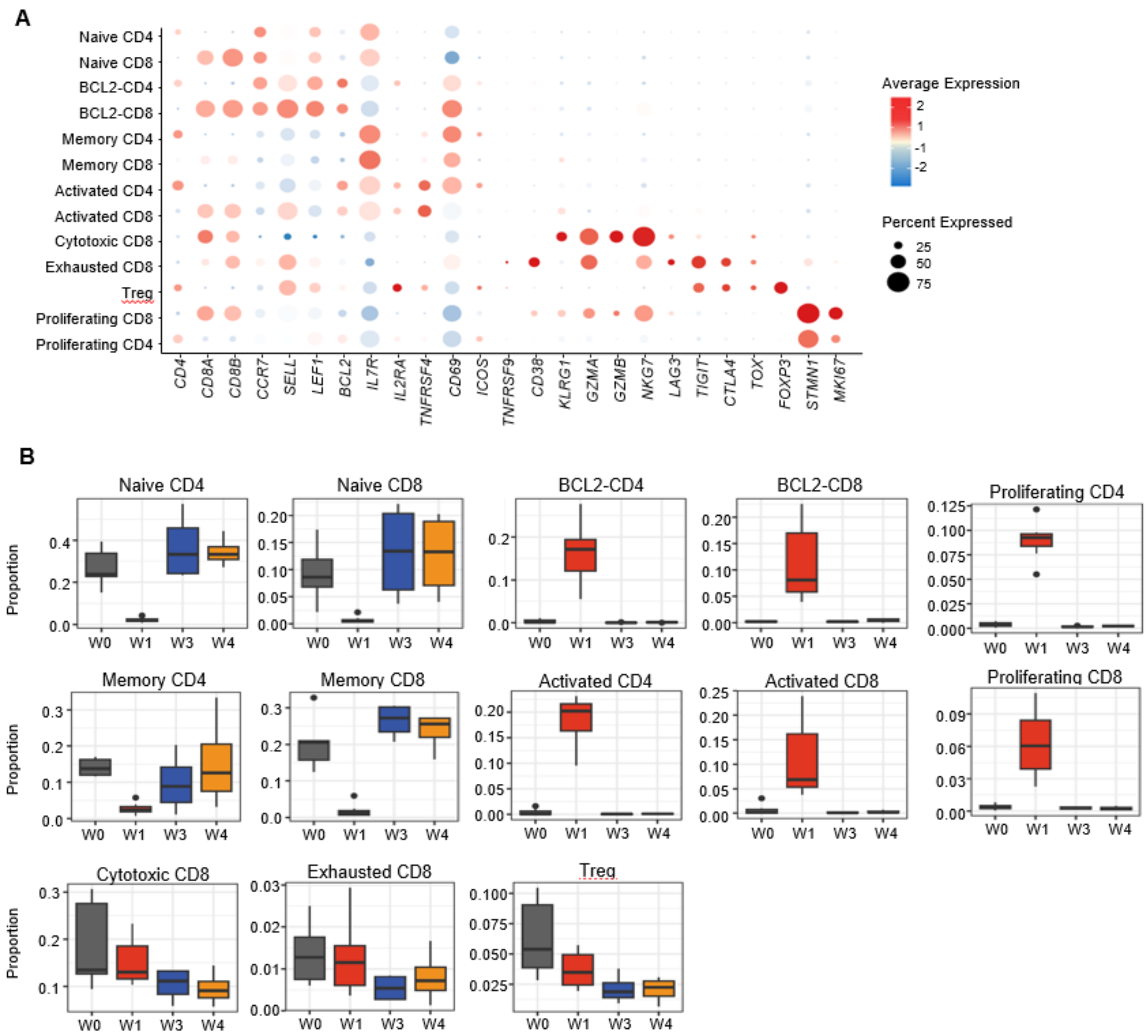

Supplementary Figure 3 Cluster annotation and cell composition. **(A)** Expression of marker genes used to identify CD4⁺ or CD8⁺ T-cell clusters. **(B)** Proportion of each cluster across time-points.

Supplementary Figure 4

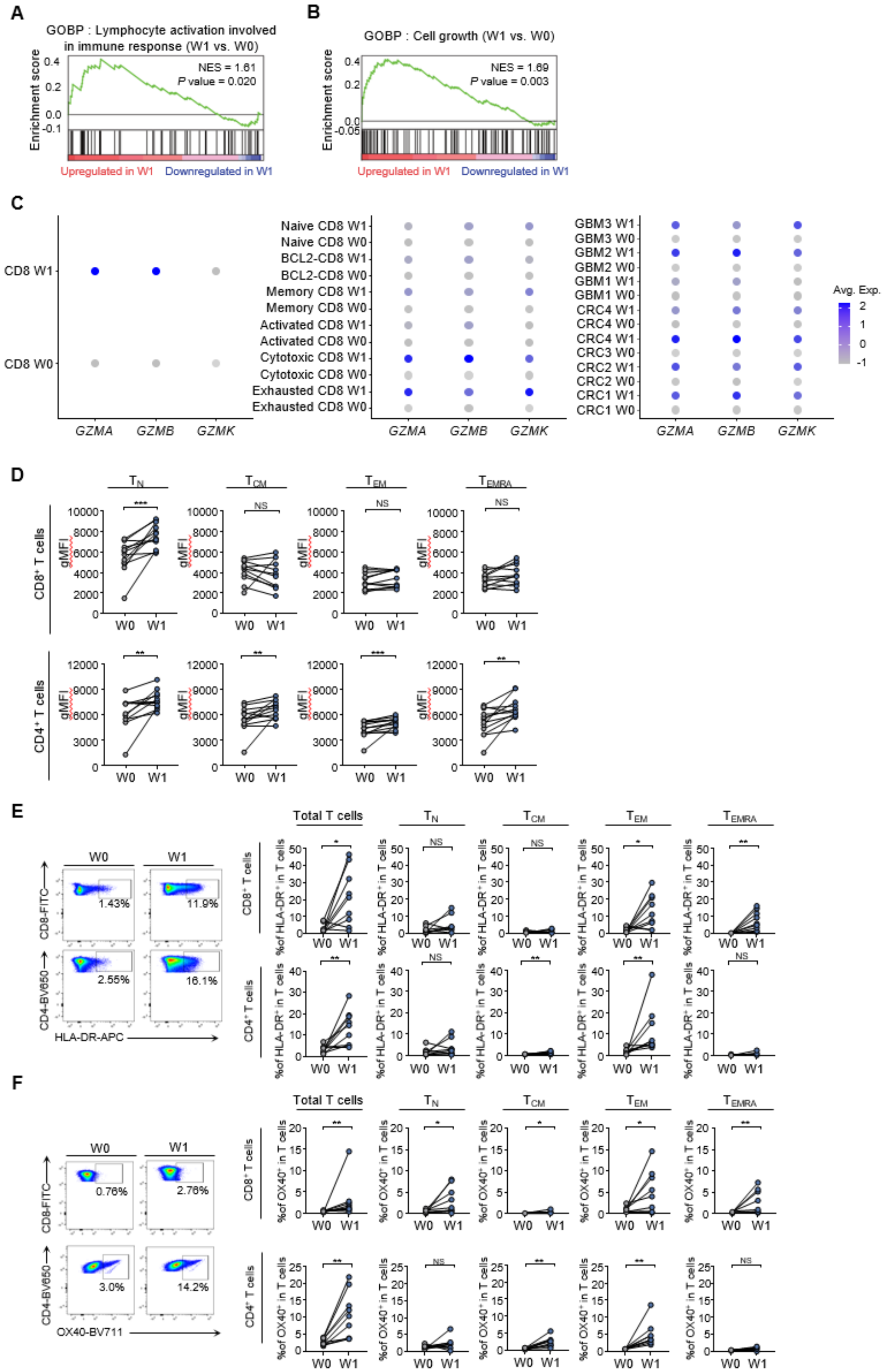

Supplementary Figure 4 Transcriptome changes induced by rhIL-7-hyFc. **(A-B)** Gene set enrichment analysis comparing W1 and W0 for GO biological processes: lymphocyte activation involved in immune response (A) and cell growth (B). **(C)** Cytotoxic gene expression changes after first administration across all $CD8^+$ T cells, per cluster, and per patient. **(D)** BCL2 expression of $CD8^+$ (upper) and $CD4^+$ (lower) T-cell subsets. **(E-F)** Frequencies of T cells expressing $HLA\text{-}DR^+$ (E) and $OX\text{-}40^+$ (F) measured by flow cytometry. Paired Wilcoxon tests were used for statistical comparisons and p value calculations. NS, Not Significant. *$p<0.05$, **$p<0.01$, ***$p<0.001$, and ****$p<0.0001$.

Supplementary Figure 5

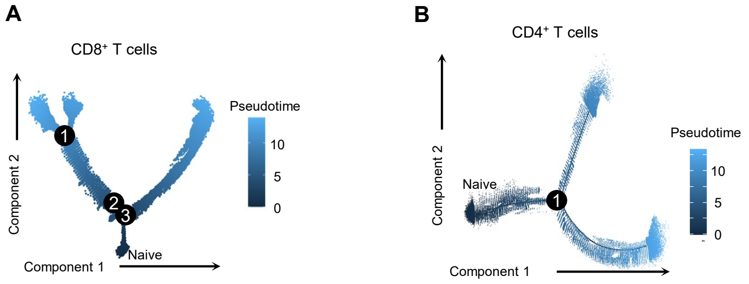

Supplementary Figure 5 Pseudotime-based state distribution of T cells. The pseudotime for $CD8^+$ (A) and $CD4^+$ (B) T cells are plotted. Psuedotime was set to start at Naive cell cluster.

Supplementary Figure 6

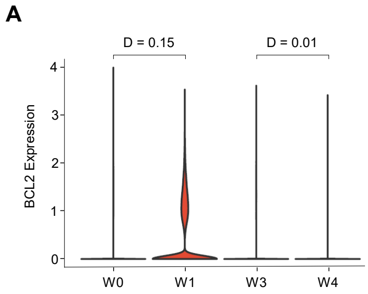

Supplementary Figure 6 *BCL2* expression across time-points. Distribution of *BCL2* expression in scRNA-seq across W0, W1, W3, and W4. Statistical comparisons were conducted with Kolmogorov-Smirnov D statistics.

Supplementary Figure 7

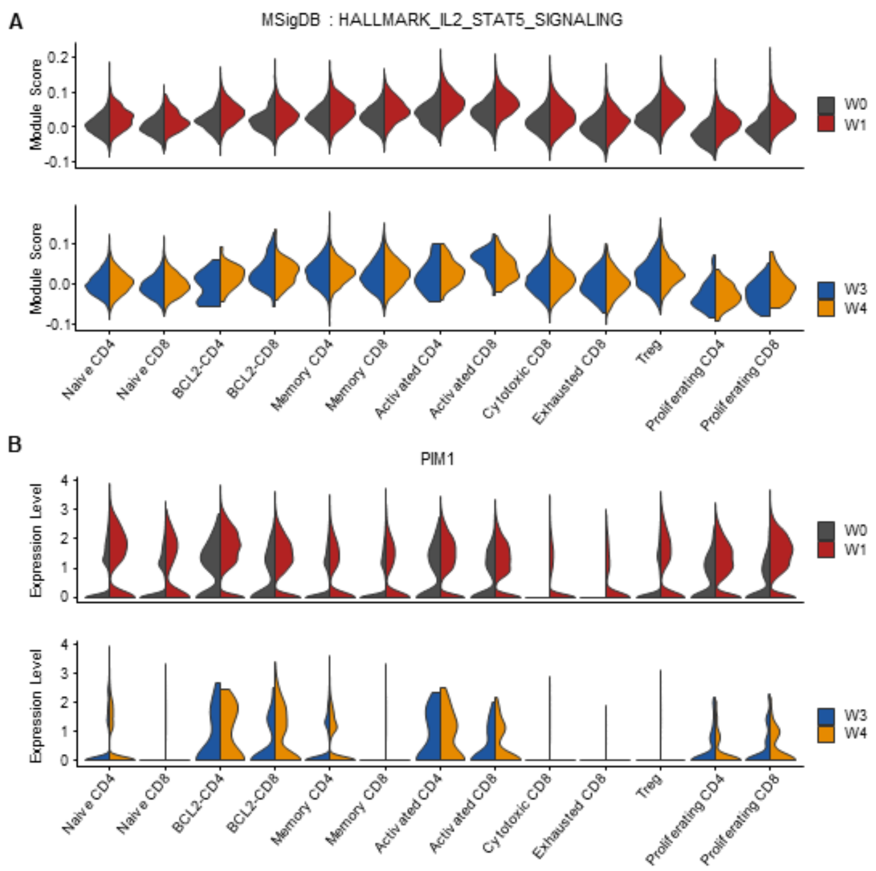

Supplementary Figure 7 Single-cell module scores for the MSigDB Hallmark IL-2/STAT5 signaling gene set (A) and *PIM1* expression (B) across T cell clusters comparing pre- and post-treatment timepoints for the first dose (W0 vs W1, top) and second dose (W3 vs W4, bottom).

Supplementary Table 1. The patients' demographic and clinical characteristics

Patient ID	Cancer Type	Stage	Prior therapy	rhIL-7-hyFc dose (μg/kg)	scRNA-seq	Flow cytometry
C1010	Sarcoma	Stage IV	surgery, radiation, chemotherapy	60		V
C1020	Breast cancer	Stage IV	chemotherapy	60		V
C1030	Colorectal cancer	Stage IV	surgery, chemotherapy	60		V
C2010	Colorectal cancer	Stage IV	surgery, chemotherapy	120		V
C2020	Ovarian cancer	Stage IV	surgery, radiation, chemotherapy	120		V
C2030	Colorectal cancer	Stage IV	surgery, radiation, chemotherapy	120		V
C3010	Colorectal cancer	Stage IV	surgery, chemotherapy	240		V
C3020	Colorectal cancer	Stage IV	surgery, radiation, chemotherapy	240		V
C3030	Colorectal cancer	Stage IV	surgery, chemotherapy	240		V
C4010	Breast cancer	Stage IV	surgery, radiation, chemotherapy	480		V
C4020	Colorectal cancer	Stage IV	surgery, radiation, chemotherapy	480		V
C4030	Colorectal cancer	Stage IV	surgery, chemotherapy	480		V
C5010	Colorectal cancer	Stage IV	surgery, radiation, chemotherapy	720	V	V
C5020	Colorectal cancer	Stage IV	surgery, chemotherapy	720		V
C5040	Colorectal cancer	Stage IV	surgery, chemotherapy	720		V
C5050	Ampullary cancer	Stage IV	surgery, radiation, chemotherapy	720		V
C6010	Colorectal cancer	Stage IV	surgery, radiation, chemotherapy	960		V
C6020	Colorectal cancer	Stage IV	surgery, chemotherapy	960		V
C6030	Colorectal cancer	Stage IV	surgery, chemotherapy	960		V
C7010	Colorectal cancer	Stage IV	surgery, chemotherapy	1,200	V	V
C7020	Colorectal cancer	Stage IV	surgery, chemotherapy	1,200		V
C7030	Colorectal cancer	Stage IV	surgery, radiation, chemotherapy	1,200	V	V
C7040	Colorectal cancer	Stage IV	surgery, radiation, chemotherapy	1,200		V
C7060	Ovarian cancer	Stage IV	surgery, chemotherapy	1,200		V
C7050	Ovarian cancer	Stage IV	surgery, chemotherapy	1,200		V
C7120	Small bowel cancer	Stage IV	surgery, chemotherapy	1,200		V
C7130	Colorectal cancer	Stage IV	surgery, chemotherapy	1,200		V
C7140	Colorectal cancer	Stage IV	surgery, chemotherapy	1,200		V
C7150	Breast cancer	Stage IV	surgery, radiation, chemotherapy	1,200		V
C7160	Colorectal cancer	Stage IV	surgery, chemotherapy	1,200		V
C8010	Colorectal cancer	Stage IV	surgery, chemotherapy	1,700		V
C8020	Colorectal cancer	Stage IV	surgery, radiation, chemotherapy	1,700	V	V
S0107	Glioblastoma	Grade IV	surgery, concurrent chemoradiotherapy	840		V
S0202	Glioblastoma	Grade IV	surgery, concurrent chemoradiotherapy	360	V	
S0203	Glioblastoma	Grade IV	surgery, concurrent chemoradiotherapy	600		V
S0205	Glioblastoma	Grade IV	surgery, concurrent chemoradiotherapy	1,440	V	V
S0302	Glioblastoma	Grade IV	surgery, concurrent chemoradiotherapy	840	V	V
S0303	Glioblastoma	Grade IV	surgery, concurrent chemoradiotherapy	840		V
